# Dietary habits and obesity indices in patients with gastro-esophageal reflux disease: a comparative cross-sectional study

**DOI:** 10.1186/s12876-017-0699-1

**Published:** 2017-11-28

**Authors:** Omid Eslami, Mansour Shahraki, Ali Bahari, Touran Shahraki

**Affiliations:** 1 Department of Nutrition, School of Public Health, Iran University of Medical Sciences, Tehran, Iran; 2 Department of Nutrition, Faculty of Medicine & Children and Adolescents Health Research Center, Resistant Tuberculosis Institute, Zahedan University of Medical Sciences, P.O. Box :98167-4315, Zahedan, Iran; 3 Gastroenterology and Hepatology Research Center, Mashhad University of Medical Sciences, Mashhad, Iran; 4 Department of Pediatrics, Faculty of Medicine & Children and Adolescents Health Research Center, Resistant Tuberculosis Institute, Zahedan University of Medical Sciences, Zahedan, Iran

**Keywords:** Gastro-esophageal reflux disease, Dietary habits, Central obesity, Waist circumference

## Abstract

**Background:**

Gastro-esophageal reflux disease (GERD) is suggested to be associated with some socio-demographic and lifestyle factors. Although the roles of some factors such as obesity are well documented, evidence on the impact of other factors such as dietary habits are still inconclusive. The aim of this study was to determine the relationship between socio-demographic and lifestyle factors with GERD in participants referred to a teaching hospital in Zahedan, South-East of Iran.

**Methods:**

This comparative cross-sectional study was conducted during 2014–2015. All patients completed a structured questionnaire regarding information on socio-demographic status, lifestyle factors and dietary habits. Anthropometric indices including body mass index (BMI) and waist circumference (WC) were used to determine general and central obesity, respectively. Data were analyzed using SPSS software version 22. Value of *p* < 0.05 was considered as statistically significant.

**Results:**

Five hundred and five participants, including 285 GERD and 220 Non-GERD participants participated in the study. In univariate analysis, being married (OR = 1.57, 95%CI = 1.04, 2.36), general obesity (OR = 1.77, 95%CI = 1.11, 2.81), central obesity (OR = 2.09, 95%CI = 1.46,3.01) and consumption of citrus fruits between meals (OR = 1.69, 95%CI = 1.04, 2.73) were associated with higher odds of GERD, while higher educational level (OR = 0.53, 95%CI = 0.36,0.77) and regular physical activity ≥2 h/week (OR = 0.53, 95%CI = 0.30, 0.94) were associated with lower odds of GERD. In the adjusted model, central obesity (OR = 1.88, 95%CI = 1.18, 3.01) and consumption of citrus fruits between meals (OR = 2.22, 95%CI = 1.30, 3.81) were positively associated with odds of GERD, while higher educational level (OR = 0.55, 95%CI = 0.33, 0.91) was associated with decreased odds of GERD.

**Conclusion:**

According to the results of the current study, central obesity as determined by WC and citrus fruit intake were independent factors associated with GERD. Therefore, lifestyle modification might have a positive effect in the treatment of GERD in an urban population of Iran.

**Electronic supplementary material:**

The online version of this article (10.1186/s12876-017-0699-1) contains supplementary material, which is available to authorized users.

## Background

Gastro-esophageal reflux disease (GERD) is one of the most common disorders of the upper digestive tract worldwide. The prevalence of GERD is estimated within the range of 18.1% to 27.8% in North America, 8.8% to 25.9% in Europe, and 8.7% to 33.1% in the Middle East [1]. In Iran, prevalence of GERD is estimated within the range of 1.9 to 52% [2]. GERD is characterized primarily based on typical symptoms of heartburn and regurgitation. In addition to the esophageal manifestations of GERD, its effect on quality of life as well as extra-esophageal presentations such as dry cough, chest pain, sleep difficulties, and teeth decays, have been increasingly recognized among gastroenterologists [3, 4]. Up to now, a combination of lifestyle modifications and anti-reflux medications are recommended for the management of GERD symptoms [5].

There is evidence that several socio-demographic and lifestyle factors are predictors of GERD development. The roles of some risk factors such as obesity are well known in epidemiological studies [6]. However, evidence on other variables particularly dietary habits are still inconclusive. Current guidelines recommend that reflux-triggering foods such as coffee, chocolate, spicy foods, and citrus fruit should not be routinely eliminated in management of GERD [7]. However, some studies revealed that specific dietary choices were related to higher risk of GERD development or the severity of the disease [8, 9]. In recent years, increased awareness of the side effects of medication on the general population shifted attention gradually, from pharmacological therapies to dietary modifications as the first-line management for GERD [10, 11]. Besides, it is proposed that eating habits may contribute to GERD risk through their effect on body weight [12]. Unfavorable eating patterns, which are mainly characterized by frequent consumption of carbonated drinks, fast foods, and large portion-size meals are positively associated with development of obesity [13] which in turn, may contribute to increased GERD risk.

Although, there are many studies in Iran investigating predictors of GERD risk in terms of lifestyle related factors such as smoking and obesity, evidence on the association between dietary habits and GERD are scarce and even inconsistent. A study from North of Iran showed a positive association between lying down after meals, with increased frequency of GERD, however such an association was not found for consumption of beverages and spicy foods [14]. Similarly, a study in South of Iran found no significant association between consumption of tea, coffee, and fast foods, with GERD [15]. High burden of disease in the Iranian population [16], makes it necessary to identify factors associated with GERD in different regions, for implementing prevention programs. Therefore, the aim of the present study was to determine independent factors associated with GERD in patients referred to a teaching hospital in Zahedan, South-East of Iran.

## Methods

### Participants

A comparative cross-sectional study was conducted during 2014–2015 at Zahedan, center of Sistan and Baluchestan province in South-East of Iran, which has a referral Gastroenterology and Hepatology clinic in Ali-Ibn-AbiTalib Hospital. During the clinical interview, a gastroenterologist asked patients about the symptoms of GERD. All participants (20 to 60 years old) with frequent (more than weekly) typical reflux symptoms including heartburn and acid regurgitation with diagnosis of GERD were included in the study. In addition, 220 healthy age- and sex-matched participants with no GERD-related symptoms were regarded as the control group. The control group was chosen from among the relatives of patients who were referred to the gastroenterology unit and other medical specialties clinics in the hospital. Those with a history of upper GI surgery, taking non-steroidal anti-inflammatory drugs (NSAIDs) or anti-reflux therapies in the previous month, and participants with history of other gastrointestinal diseases were excluded. All participants signed a written informed consent. The study protocol was approved by the Ethics Committee of Children and Adolescent Health Research Center, Zahedan University of Medical Sciences, Zahedan, Iran.

### Questionnaire and anthropometric measurements

A structured questionnaire was completed by researchers of the study through face-to-face interviews (Additional file 1: The structured questionnaire was developed to obtain information on socio-demographic and lifestyle-related factors in this study.**)**. The first part of the questionnaire consisted socio-demographic information including age, sex, marital status, education, job level, and presence of any concomitant disease (asthma, diabetes, hypertension, hyperlipidemia, chronic kidney disease and/or chronic artery disease. The second part included questions about lifestyle factors including smoking (cigarette or hookah) and substance abuse (opium or nass, which is a combination of tobacco, ash, and lime), physical activity, consumption of large portion-size meals, fast foods (including sausage, fried chicken, pizza, hamburger, French fries, and doughnuts), spicy foods (including chili pepper, turmeric, ginger, cinnamon, and curry), chocolate, tea, coffee, citrus fruit, carbonated drinks, number of meals consumed daily, time interval between the last meals and sleep, and resting immediately after main meals. To assess consumption of large portion-size meals, food photographs were applied. Each photograph was comprised of foods that were habitually consumed as a main meal (breakfast, lunch, and dinner) in Iranian culture, for example, a photograph of a dish containing “rice, meat, and beans” typically represented a meal for lunch or dinner meal, served by the Iranian population. Food items were presented in their standard portion size which had been proposed by the USDA food guide pyramid (for example one cup, slice, or teaspoon) [17]. Then, participants were asked to estimate whether they consumed a larger portion than what was presented in the past week, and if they responded “yes,” the frequency of consumption was assessed. An intake of large portion-size meals for each participant was recorded by summing up the weekly consumption of these meals.

A trained staff conducted anthropometric measurements. Weight and height were measured with accuracy of 0.1 kg and 0.5 cm respectively, using Seca instruments (Seca, Hamburg, Germany). Body mass index (BMI) was calculated as weight (kg)/height^2^ (m^2^). Waist circumference (WC) was measured by a non-elastic tape at the midpoint of the last rib margin and iliac crest according to WHO standards [18]. Values of BMI ≥ 30 kg/m^2^ was defined as general obesity. Also, WC ≥102 cm in men and WC ≥ 88 cm in women were regarded as central obesity.

### Statistical analysis

The Chi-square test was used to assess the differences in socio-demographic and lifestyle factors between GERD and non-GERD participants. Multiple logistic regression model was employed to detect independent factors associated with GERD. Validity of the regression model was checked using the Hosmer–Lemeshow goodness-of-fit test. Multicollinearity between variables in the model was evaluated using the Variance Inflation Factor (VIF). Variables were excluded from the adjusted model if they had showed multicollinearity, which was considered as VIF > 5 [19]. Results were expressed as percentages, crude and adjusted odds ratios (ORs), and 95% confidence intervals (CIs). Data were analyzed using SPSS software version 22 (IBM Corp., Armonk, NY, USA). A value of *p* < 0.05 was considered as statistically significant.

## Results

A total of 505 participants consisting of 285 GERD and 220 Non-GERD cases were included. Table 1 compares socio-demographic status and obesity indices between GERD and Non-GERD participants. In comparison with the Non-GERD group, the GERD group had significantly higher percentage of married individuals (*p* = 0.02) as well as the presence of concomitant disease (*p* = 0.001). Level of education (p = 0.001) and regular physical activity ≥2 h per week (*p* = 0.03) inversely influenced the prevalence of GERD. In addition, the prevalence of general obesity (*p* = 0.01) and central obesity (p = <0.001) were significantly higher in the GERD group compared to the Non-GERD ones. Dietary habits of participants are presented in Table 2. There were no significant differences in dietary habits between the two groups.

**Table 1:** The association between socio-demographic factors and obesity indices with GERD in study participants

Variable	Total *N* = 505		GERD *N* = 285		Non- GERD *N* = 220		*P*-value*
	N	%	N	%	N	%	
Age (years)							
< 35	292	57.8	160	56.1	132	60	0.38
≥ 35	213	42.2	125	43.9	88	40	
Gender							
Men	156	30.9	78	27.4	78	35.5	0.051
Women	349	69.1	207	72.6	142	64.5	
Marital status							
Single	123	24.4	59	20.7	64	29.1	0.02**
Married	382	75.6	226	79.3	156	70.9	
Education (years)							
< 12 years schooling	328	65	203	71.2	125	56.8	0.001**
≥ 12 years schooling	177	35	82	28.8	95	43.2	
Job							
Unemployed	337	66.7	198	69.5	139	63.2	0.32
Self-employed	97	19.2	51	17.9	46	20.9	
Employee	71	14.1	36	12.6	35	15.9	
Smoking or substance abuse							
Yes	61	12.1	37	13.0	24	10.9	0.47
No	444	87.9	248	87.0	196	89.1	
Physical activity (hours/wk)							
< 2	451	89.3	262	91.9	189	85.9	0.03**
≥ 2	54	10.7	23	8.1	31	14.1	
Concomitant diseases ^a^							
Yes	165	32.7	111	38.9	54	24.5	0.001**
No	340	67.3	174	61.1	166	75.5	
BMI							
< 30	407	80.6	219	76.8	188	85.5	0.01**
≥ 30	98	19.4	66	23.2	32	14.5	
WC (cm)							
Men <102; Women <88	272	53.9	131	46.0	141	64.1	<0.001**
Men ≥102; Women ≥88	233	46.1	154	54.0	79	35.9	

*BMI* body mass index, *WC* waist circumference. ^a^Concomitant diseases, including asthma, diabetes, hypertension, hyperlipidemia, chronic kidney disease and/or chronic artery disease. *p-values was derived from Chi-square test. **Significant at the level of *p*< 0.05

**Table 2:** The association between dietary habits and GERD in study participants

Variable	Total *N *= 505		GERD *N *= 285		Non- GERD *N *= 220		*P*-value*
	N	%	N	%	N	%	
Numbers of meals consumed daily(meals/d)							
< 3	185	36.6	107	37.5	78	35.5	0.62
≥ 3	320	63.4	178	62.5	142	64.5	
Time interval between the last meal and sleep (hours)							
< 2	247	48.9	146	51.2	101	45.9	0.23
≥ 2	258	51.1	139	48.8	119	54.1	
Resting immediately after the main meals							
Yes	335	66.3	186	65.3	149	67.7	0.56
No	170	33.7	99	34.7	71	32.3	
Large portion-size meals (times/wk)							
< 2	236	46.7	123	43.2	113	51.4	0.06
≥ 2	269	53.3	162	56.8	107	48.6	
Fast foods intake (times/wk)							
< 2	468	92.7	267	93.7	201	91.4	0.32
≥ 2	37	7.3	18	6.3	19	8.6	
Spicy foods intake (times/wk)							
< 2	271	53.7	152	53.3	119	54.1	0.86
≥ 2	234	46.3	133	46.7	101	45.9	
Chocolate intake (times/wk)							
< 2	324	64.2	179	62.8	145	65.9	0.47
≥ 2	181	35.8	106	37.2	75	34.1	
Tea intake							
None	38	7.5	20	7	18	8.2	0.54
Just before meals	109	21.6	67	23.5	42	19.1	
Just after meals	98	19.4	51	17.9	47	21.4	
Between meals	260	51.5	147	51.6	113	51.4	
Coffee intake							
None	474	93.9	272	95.4	202	91.8	0.18
Just before OR after meals	12	2.4	4	1.4	8	3.6	
Between meals	19	3.8	9	3.2	10	4.5	
Citrus intake							
None	360	71.3	195	68.4	165	75	0.08
Just before OR after meals	52	10.3	28	9.8	24	10.9	
Between meals	93	18.4	62	21.8	31	14.1	
Carbonated drinks intake							
None	217	43	127	44.6	90	40.9	0.16
Within meals	234	46.3	134	47	100	45.5	
Between meals	54	10.7	24	8.4	30	13.6	

*p-values was derived from Chi-square test

Table 3 shows results of univariate and multiple regression analysis for detection of independent factors associated with GERD development, as the study outcome. In univariate analysis, being married (OR = 1.57, 95%CI = 1.04, 2.36), general obesity (OR = 1.77, 95%CI = 1.11, 2.81), central obesity (OR = 2.09, 95%CI = 1.46, 3.01), and consumption of citrus fruits between meals (OR = 1.69, 95%CI = 1.04, 2.73) were significantly associated with higher odds of GERD, while higher level of education (OR = 0.53, 95%CI = 0.36, 0.77) and regular physical activity ≥2 h/week (OR = 0.53, 95%CI = 0.30, 0.94) were associated with lower odds of GERD. Based on the Hosmer-Lemeshow goodness-of-fit test, the regression model showed a good calibration (Chi-square 7.52; degrees of freedom = 8; *p*-value = 0.48). Also, no sign of multicollinearity was observed between variables, as all variables had VIF ≤ 2 and thus, all independent variables were included in the regression model. In the multiple regression model, central obesity (OR = 1.88, 95%CI = 1.18, 3.01) and consumption of citrus fruits between meals (OR = 2.22, 95%CI = 1.30, 3.81) were associated with increased odds of GERD, while higher level of education (>12 years) (OR = 0.55, 95%CI = 0.33, 0.91) was associated with decreased odds of GERD.

**Table 3:** Factors related to GERD development as the outcome variable by univariate analysis and multiple regression analysis (*N* = 505)

Variable	B	Univariate OR			Adjusted OR		
			95% CI			95% CI	
		OR	Lower	Upper	OR	Lower	Upper
Age (years)							
< 35^a^	−0.30	1			1		
≥ 35		1.17	0.82	1.67	0.734	0.466	1.15
Gender							
Men^a^	0.02	1			1		
Women		1.45	0.99	2.13	1.02	0.53	1.97
Marital status							
Single^a^	0.19	1			1		
Married		1.57*	1.04	2.36	1.21	0.75	1.97
Education (years)							
< 12 years schooling^a^	−0.59	1			1		
≥ 12 years schooling		0.53*	0.36	0.77	0.55*	0.33	0.91
Job							
Unemployed^a^		1			1		
Self-employed	0.07	0.77	0.49	1.22	1.08	0.52	2.22
Employee	0.19	0.72	0.43	1.20	1.21	0.57	2.53
Smoking or substance abuse							
No^a^	0.33	1			1		
Yes		1.21	0.70	2.10	1.40	0.76	2.56
Physical activity (h/wk)							
< 2^a^	−0.38	1			1		
≥ 2		0.53*	0.30	0.94	0.67	0.34	1.32
BMI (kg/m^2^)							
< 30^a^	0.27	1			1		
≥ 30		1.77*	1.11	2.81	1.32	0.76	2.29
WC (cm)							
Men <102; Women <88^a^	0.63	1			1		
Men ≥102; Women ≥88		2.09*	1.46	3.01	1.89*	1.18	3.01
Numbers of meals consumed daily (meals/d)							
< 3^a^	−0.16	1			1		
≥ 3		0.91	0.63	1.31	0.85	0.56	1.27
Time interval between the last meal and sleep (hours)							
< 2^a^	−0.16	1			1		
≥ 2		0.80	0.56	1.15	0.84	0.57	1.24
Resting immediately after the main meals							
No^a^	0.004	1			1		
Yes		0.89	0.61	1.30	1.004	0.66	1.51
Large portion-size meals (times/wk)							
< 2^a^	0.32	1			1		
≥ 2		1.39	0.97	1.98	1.38	0.94	2.04
Fast foods intake (times/wk)							
< 2^a^	−0.13	1			1		
≥ 2		0.71	0.36	1.39	0.87	0.42	1.82
Spicy foods intake (times/wk)							
< 2^a^	−0.02	1			1		
≥ 2		1.03	0.72	1.46	0.97	0.66	1.45
Chocolate intake (times/wk)							
< 2^a^	0.19	1			1		
≥ 2		1.14	0.79	1.65	1.21	0.80	1.82
Tea intake							
None^a^		1			1		
Just before meals	0.14	1.43	0.68	3.02	1.16	0.51	2.60
Just after meals	−0.15	0.97	0.46	2.06	0.85	0.37	1.94
Between meals	−0.03	1.17	0.59	2.31	0.96	0.45	2.04
Coffee intake							
None^a^		1			1		
Just before/ after meals	−0.53	0.37	0.11	1.25	0.58	0.15	2.18
Between meals	−0.19	0.66	0.26	1.67	0.82	0.29	2.3
Citrus intake							
None^a^		1			1		
Just before/after meals	0.35	0.98	0.55	1.76	1.42	0.73	2.76
Between meals	0.80	1.69*	1.04	2.73	2.22*	1.30	3.81
Carbonated drinks intake							
None^a^		1			1		
Within meals	−0.07	0.95	0.65	1.38	0.92	0.61	1.39
Between meals	−0.57	0.56	0.31	1.03	0.56	0.28	1.10

*BMI* body mass index, *WC* waist circumference, *OR* odds ratio, *CI* confidence interval

^a^Reference group. *Significant at the level of *p* < 0.05

## Discussion

The aim of the present study was to assess dietary habits, lifestyle, and socio-demographic status of GERD patients in South-East of Iran. Although central obesity and citrus fruit intake were independent factors associated with GERD, such an association was not found for other dietary habits, BMI, and socio-demographic status. Previous researches have indicated that high BMI, an index of general obesity, is a risk factor for GERD development and severity of symptoms [20, 21]. In this study, central obesity, determined by high WC was an independent predictor of GERD. Although high BMI was associated with higher odds of GERD in the univariate analysis, this association remained non-significant in the adjusted model analysis. Excess abdominal fat can increase the risk of GERD through several mechanisms including increased intra-gastric pressure, decreased lower esophageal sphincter (LES) pressure, and increased frequency of transient lower esophageal sphincter relaxation (TLSR), which results in acid reflux [22]. Similarly, a study on 2457 GERD patients reported that central obesity as determined by WC was one of risk factors inducing erosive esophagitis [23]. Another study by Crews et al. reported that central obesity based on high values of waist-to-hip ratio (WHpR), was an independent risk factor of erosive esophagitis [24]. It should be noted that differences in obesity indices in predicting GERD risk have been reported in other chronic disorders. A previous study found that central obesity determined by high WC was a strong predictor of adverse cardiometabolic profile, compared to BMI or WHpR [25]. In contrast, Gunji et al. found that BMI was a predictor of erosive esophagitis, while such an association was not found for central obesity measured by visceral adipose tissue and waist girth [26]. Moreover, some studies have shown that general obesity is a risk factor for severity of GERD symptoms alongside its development. However, it should be noted that the prevalence of general obesity was lower than that of central obesity in our study (19.4% vs. 46.1%), which may be suggestive of the differences between the BMI in our study compared with that of others.

The association between diet and GERD has been an issue of interest in epidemiological studies. In the present study, citrus fruit intake was the only dietary factor that might be associated with higher odds of GERD. Up to now, results on diet-GERD relationship are inconsistent among various studies. Several studies reported that consumption of caffeinated beverages (coffee and tea), carbonated drinks, citrus fruit, chocolate, spicy foods, and large-volume meals, were related to higher risk of GERD or triggering symptoms [8, 9, 27–29]. It is proposed that these foods can decrease LES pressure and/or slow gastric emptying which may result in development of GERD symptoms [27]. In addition, the association between coffee and GERD is still unclear. Shimamoto et al. found no association between coffee intake and GERD among 8103 healthy Japanese individuals [30]. Moreover, results of a meta-analysis reported no association between coffee intake and GERD [31]. In our study, about 90% of participants did not consume coffee. Therefore, it seems that any possible relationship between coffee and GERD may not be obvious in this study. In terms of tea consumption and GERD, several epidemiological surveys had regarded tea as a risk factor of GERD [27, 32], although, this relationship varied depending on the type of tea consumed, such as green tea, strong tea or peppermint tea [27, 33, 34], as well as quantity of tea intake [32]. In this study, no significant association was found between the timing of tea intake and risk of GERD, which is in line with the finding of previous epidemiological reports [35, 36]. Also, we found no association between carbonated beverages and GERD. Similarly, a systematic review demonstrated that carbonated beverages were neither related to GERD development, nor exacerbation of GERD complication [37]. It should be noted that in this study, the amount of the liquid intake was not recorded. Some studies have shown that intake of large volumes of liquid may be associated with GERD development or disease severity [8, 28]. Moreover, it is proposed that this association may vary, depending on whether or not the liquid is consumed with meals. Accordingly, a study reported that intake of coffee alone had no significant changes on LES pressure, while consumption of coffee after a lunch meal significantly reduced LES pressure [38]. However, this topic seems to be still inconclusive and more researches with an emphasis on both quantity and timing of liquid intake are needed.

In terms of socio-demographic status and GERD, being married was associated with higher odds of GERD in univariate analysis. While, higher educational level was associated with lower odds of GERD in both univariate and adjusted analysis. Similarly, previous studies reported lower educational level or being married as risk factors of GERD [39, 40]. It has been suggested that the above-mentioned factors may be indirectly related to GERD through their effects on lifestyle conditions including eating habits, psychosocial stress, and hygiene practices [41]. However, other studies have demonstrated otherwise [42, 43]. It should be noted that this association could not be simply explained due to this cross-sectional design, warranting further researches in this field.

Another concern is the lack of a clear association between smoking and GERD. While previous studies have indicated smoking as an independent predictor of GERD development [21, 26, 29, 44], others could not confirm such an association [24, 35, 40], in the present study, smoking was not associated with GERD development. This is inconsistent with findings of the study conducted by Esmaillzadeh et al., which showed that in Iranian adult men, smoking was not a significant predictor for GERD [45]. A similar population-based cohort study in Norwegian population clarified that cessation of smoking was associated with improvement of GERD from severe to no or minor complaints, only among individuals within normal range of BMI but not among overweight individuals [46]. Although, smoking has a stimulatory effect on acid exposure, only 4% of the variations in GERD symptoms are attributed to acid exposure [47, 48]. Besides, the inconsistency among studies might be due to differences in the socio-demographic status of study populations, sample sizes, criteria for diagnosis of GERD, and definition of smoking status. Of note is that in this study, similar to that of Esmaillzadeh et al. (7.8% of the study participants were smokers) [45], prevalence of smoking was low, making it difficult to draw a significant relationship between smoking with GERD.

There are some limitations of our study, worth mentioning. First, we cannot ignore the effect of recall bias on our investigation, although trained staff were responsible for data collection, to reduce any possible errors which might be influenced in the results. Second, dietary intake from other food groups such as dairy products, meats, grains, and fats, as well as total energy and nutrient intake were not investigated. A study by Wu et al. found that intake of meat, oil, salt, and calcium were related to an increased risk of GERD, while intake of carbohydrate, protein, vitamin C, starches, egg, and fruits were inversely associated with GERD development [49]. However, the present study focused on those specific dietary sources which have mostly known for their refluxogenic effect. We suggest that future studies should focus on the diet-GERD relationship in the context of dietary patterns, rather than of specific nutrients, food items, or food groups. This approach therefore, will assess the combined effect of various dietary components, and as a result, provide a more comprehensive and precise information on the diet-GERD relationship.

## Conclusions

To our knowledge, this study provides the first evidence on predictors of GERD in the Sistan and Baluchistan province. Central obesity as determined by WC and citrus fruit intake were independent factors associated with GERD development in the study population. Due to increasing prevalence of GERD in Iran, detection of risk factors for GERD can assist health professional to promote preventive programs in this region.

## Additional file

Additional file 1: The structured questionnaire was developed to obtain information on socio-demographic and lifestyle-related factors in this study. (DOCX 16 kb)

## Abbreviations

BMI: Body mass index
CIs: Confidence intervals
GERD: Gastro-esophageal reflux disease
GI: Gastrointestinal
LES: Lower esophageal sphincter
NSAIDs: Non-steroidal anti-inflammatory drugs
ORs: Odds ratios
TLSR: Transient lower esophageal sphincter relaxation
USDA: US Department of Agriculture
WC: Waist circumference
WHO: World Health Organization
WHpR: Waist-to-hip ratio
