# Supplementary material for: Dietary habits and obesity indices in patients with gastro-esophageal reflux disease: a comparative cross-sectional study
Source: BMC Gastroenterol. 2017 Nov 28;17:132. doi: 10.1186/s12876-017-0699-1 (PMC5704630; doi:10.1186/s12876-017-0699-1)
Supplement: Additional file 1: — The structured questionnaire was developed to obtain information on socio-demographic and lifestyle-related factors in this study. (DOCX 16 kb) [file 12876_2017_699_MOESM1_ESM.docx]

**The Questionnaire**

***Dietary habits and obesity indices in patients with gastro-esophageal reflux disease: a comparative cross-sectional study***

| **Code** | **Last name** | **First name** | **Date** | | | | | | | |
| --- | --- | --- | --- | --- | --- | --- | --- | --- | --- | --- |
|  |  |  | **Month** | | **Day** | | **Year** | | | |
|  |  |  |  |  |  |  | 2 | 0 |  |  |

**Section A: General information**

- **Age** (years): …… 1) < 35 2) ≥ 35
- **Sex**: 1) Male 2) Female
- **Marital status**: 1) Single 2) Married
- **Education** (years): …… 1) < 12 years schooling 2) ≥ 12 years schooling
- **Job**: 1) Unemployed 2) Self-employed 3) Employee
- **Do you have any of the following concomitant diseases? If Yes, Which one?**

1) Asthma 2) Diabetes 3) Hypertension 4) Hyperlipidemia

5) Chronic kidney disease 6) Chronic artery disease 7) none

**Section B: Lifestyle related factors** (Smoking, physical activity, and eating habits)

- **Do you smoke cigarettes and/or hookah?**

1) Yes

2) No

3) I am an ex-smoker

- **Do you use opium and/or nass (a combination of tobacco, ash, and lime)?**

1) Yes

2) No

3) I am an ex-substance abuser

- **How much time in a week do you spend doing moderate to vigorous physical activities,** *such as brisk walking, pushing and lifting heavy objects*

1) < 2 hours*/week*

2) ≥ 2 hours*/week*

- **How many meals do you eat daily? Including main meals and snacks.**

1) < 3 *meals/day*

2) ≥ 3 *meals/day*

- **How long do you wait to go to the bed after eating the last meal?**

1) < 2 *hours*

1) ≥ 2 *hours*

- **Do you lie down immediately after the main meals?**

1) Yes

2) No

- **How often do you eat large portion-size meals in a week? *NOTE****: The study dietitian should investigate the portion sizes of consumed foods using the Food Album.*

1) < 2 *times/ week*

2) ≥ 2 times*/ week*

- **How often do you eat fast foods in a week?** *Including sausage, fried chicken, pizza, hamburger, French fries, and doughnuts*.

1) < 2 *times/ week*

2) ≥ 2 *times/ week*

- **How often do you eat spicy foods in a week?** *Including chili pepper, turmeric, ginger, cinnamon, and curry.*

1) < 2 *times/ week*

2) ≥ 2 *times/ week*

- **How often do you eat chocolate in a week?**

1) < 2 *times/ week*

2) ≥ 2 *times/ week*

- **Do you drink tea?**

1) Yes

2) No

- **If Yes, at what time of day do you drink tea?**

1) Just before meals

2) Just after meals

3) Between meals

- **Do you drink coffee?**

1) Yes

2) No

- **If Yes, at what time of day do you drink coffee?**

1) Just before meals

2) Just after meals

3) Between meals

- **Do you consume citrus?**

1) Yes

2) No

- **If Yes, at what time of day do you consume citrus?**

1) Just before meals

2) Just after meals

3) Between meals

- **Do you consume carbonated drinks?**

1) Yes

2) No

- **If Yes, at what time of day do you consume carbonated drinks?**

1) Within meals

2) Between meals
